# Multivariate Kalman filter regression of confounding physiological signals for real-time classification of fNIRS data

**DOI:** 10.1117/1.NPh.9.2.025003

**Published:** 2022-06-08

**Authors:** Antonio Ortega-Martinez, Alexander Von Lühmann, Parya Farzam, De’Ja Rogers, Emily M. Mugler, David A. Boas, Meryem A. Yücel

**Affiliations:** a Boston University Neurophotonics Center, Boston, Massachusetts, United States; b Berlin Institute of Technology, Machine Learning Department, Berlin, Germany; c Facebook Reality Labs Research, Menlo Park, California, United States

**Keywords:** functional near-infrared spectroscopy, classification, real-time, Kalman, brain–computer interface, regressors, time-embedded canonical correlation analysis

## Abstract

**Significance:**

Functional near-infrared spectroscopy (fNIRS) is a noninvasive technique for measuring hemodynamic changes in the human cortex related to neural function. Due to its potential for miniaturization and relatively low cost, fNIRS has been proposed for applications, such as brain–computer interfaces (BCIs). The relatively large magnitude of the signals produced by the extracerebral physiology compared with the ones produced by evoked neural activity makes real-time fNIRS signal interpretation challenging. Regression techniques incorporating physiologically relevant auxiliary signals such as short separation channels are typically used to separate the cerebral hemodynamic response from the confounding components in the signal. However, the coupling of the extra-cerebral signals is often noninstantaneous, and it is necessary to find the proper delay to optimize nuisance removal.

**Aim:**

We propose an implementation of the Kalman filter with time-embedded canonical correlation analysis for the real-time regression of fNIRS signals with multivariate nuisance regressors that take multiple delays into consideration.

**Approach:**

We tested our proposed method on a previously acquired finger tapping dataset with the purpose of classifying the neural responses as left or right.

**Results:**

We demonstrate computationally efficient real-time processing of 24-channel fNIRS data (400 samples per second per channel) with a two order of selective magnitude decrease in cardiac signal power and up to sixfold increase in the contrast-to-noise ratio compared with the nonregressed signals.

**Conclusion:**

The method provides a way to obtain better distinction of brain from non-brain signals in real time for BCI application with fNIRS.

## 1 Introduction

Functional near-infrared spectroscopy (fNIRS) is a noninvasive technique capable of monitoring brain activity in the human cortex as revealed by changes in cerebral hemodynamics.^1^^,^^2^ While fNIRS is constrained to cortical brain areas, it has comparatively less stringent technical requirements than fMRI, opening the possibility for portable applications in the everyday world.^3^ In addition, due to its immunity to electromagnetic artifacts and relative resistance to motion artifacts, fNIRS can be used in patient populations that are otherwise restricted for fMRI, such as children or people with metal implants or pacemakers.^4^ Multiple applications have been proposed for wearable fNIRS systems, for example, in the fields of neuroergonomics,^5^ brain computer interfaces (BCIs),^6^ and neuromarketing.^7^ Portable fNIRS systems will also improve the capabilities for performing basic research on the mental processes related to locomotion and social interactions.^8^^,^^9^

In a typical continuous wave (CW) fNIRS application, red or near-infrared light is used to interrogate the tissue. The backscattered light is measured a few centimeters away from the source. The changes in light intensity detected at the scalp relate to the hemodynamics of the tissue traversed by the photons. Most applications use at least two wavelengths to be able to separate the contributions to the absorption from oxyhemoglobin (HbO) and deoxyhemoglobin (HbR). CW fNIRS cannot measure absolute concentrations of the chromophores, but it can estimate changes in their concentration using the modified Beer–Lambert law (MBLL).^10^

One of the main challenges in interpreting raw CW fNIRS data comes from the potentially low contrast-to-noise ratio of the neural-specific signal due to the relatively high magnitude of other signal components, such as those generated by hemodynamic fluctuations that naturally occur in the scalp and underlying vasculature.^11^ Conventional fNIRS brain activation experiments compensate for that by having the subject perform multiple repetitions of a task, after which the statistics of the signal during the stimulation period are calculated to determine if activation likely happened. Despite this, hemodynamic changes in the extracerebral tissue temporally aligned with the stimulation are still present in the block averages and can be erroneously attributed to the brain,^12^ severely complicating single stimulus estimation of the brain response. Regression techniques, especially when combined with multimodal monitoring can be used to compensate for this.^13^ The general linear model (GLM)^14^ is a common regression analysis method used to estimate the statistics of the evoked hemodynamic responses to brain activity. It models the fNIRS signal as a linear superposition of the hemodynamic response function (HRF) and other extracerebral components (nuisance signals) plus a residual. Regression finds the least-squares estimate of the coefficients for each of the components that best explains the observed variance in the data. The more complete and accurate the model, the better the data variance can be explained and thus the underlying brain activity can be estimated.

It has been shown^15^ that modeling the nuisance signals with relevant auxiliary signals can significantly improve the final estimate of the brain activation in fNIRS. Common auxiliary measurements are short separation channels^16^ (which measure scalp hemodynamic fluctuations), blood pressure (BP),^17^ respiration (RESP), photoplethysmography (PPG), and accelerometer signals.^18^^–^^20^ Until recently, most methods that reduce physiological nuisance in fNIRS with the help of auxiliary signals as regressors did not sufficiently account for temporal delays between signals. fNIRS signals depend on local blood oxygenation and flow, which are directly affected by the heterogeneous vasculature in the human head. Therefore, noninstantaneous coupling between signals across different measurement channels—but also between fNIRS signals and other auxiliary signals—can be observed. If not accounted for, these temporal delays reduce the quality and accuracy of the regressors used in the model and thus lead to suboptimal contrast-to-noise ratio of the estimated hemodynamic brain activity. As a remedy, recent research has demonstrated the advantages of combining the auxiliary measurements into optimally delayed composite regressors using temporally embedded canonical correlation analysis (tCCA) for the regression of fNIRS signals.^21^ The authors compared the performance of several auxiliary measurements and found that short separation channels and accelerometers provide most of the discriminating power—two signals that are straightforward and cost-effective to also integrate in wearable fNIRS devices and BCI, by providing physiological and motion artifact removal.

BCI applications can benefit from providing feedback to the user, which has demonstrated to increase the classification accuracy of the decisions taken by the BCI.^22^ While fNIRS has been gaining traction in the BCI community, fNIRS signals are usually processed offline for that research, and regression techniques to separate the brain signals from the nuisances are almost never used.^6^ This is in part because the GLM is too computationally inefficient in real-time due to it requiring matrix inversions at each acquired sample. In addition, the GLM model might be ill-conditioned for single stimulus regression, depending on the number of samples processed.

The Kalman filter is a recursive algorithm that has been previously proposed for removing motion artifacts of fNIRS signals^23^ or as an alternative to the GLM to perform the HRF regression.^24^^–^^26^ Compared with the GLM, the Kalman filter can adapt to the dynamic properties of the system and is more computationally efficient, especially if using a state-space model not requiring matrix inversions. The Kalman filter also integrates knowledge of the noise of the system and priors for the regressors, allowing for optimal and efficient sample-by-sample single stimulus estimation. Previous Kalman filter literature has approached the fNIRS physiology and artifact removal either by modeling periodic physiology as sine waves^27^ or by using auxiliary measurements such as short separation channels in a balloon model,^28^ among other approaches. None of these approaches take noninstantaneous coupling between channels into consideration. Furthermore, the problem of optimal Kalman filter parameter selection, or Kalman tuning, is ignored by most fNIRS literature, and parameters are chosen in postprocessing until results are optimized. For an online application, it is necessary to have a strategy beforehand to choose the adequate parameters for the experiment being performed.

Here, we propose an implementation of the Kalman filter using multimodal tCCA regressors to be able to separate, in real-time, the contribution of the neural response from the nuisance signals and motion artifacts with implications for wearable fNIRS applications such as BCI. We implement strategies to integrate the Kalman filter with auxiliary signals generated from a head-mounted accelerometer and short separation channels combined as optimally delayed composite regressors and tune the Kalman and tCCA parameters from baseline data. We tested the real-time root mean-squared error (RMSE) performance of the filter on resting data augmented with synthetic HRFs and compared it with the performance of the GLM. We implemented and tested a real-time prerecorded regression pipeline to demonstrate the ability of performing real-time classification of finger tapping data into left versus right. Finally, we also calculated the classification performance for single wavelength fNIRS data to determine the potential loss in classification performance when using a single wavelength^29^ device for BCI applications, a strategy that could be used to further reduce the complexity of portable fNIRS instruments.^29^

## 2 Materials and Methods

**Fig. 1:**
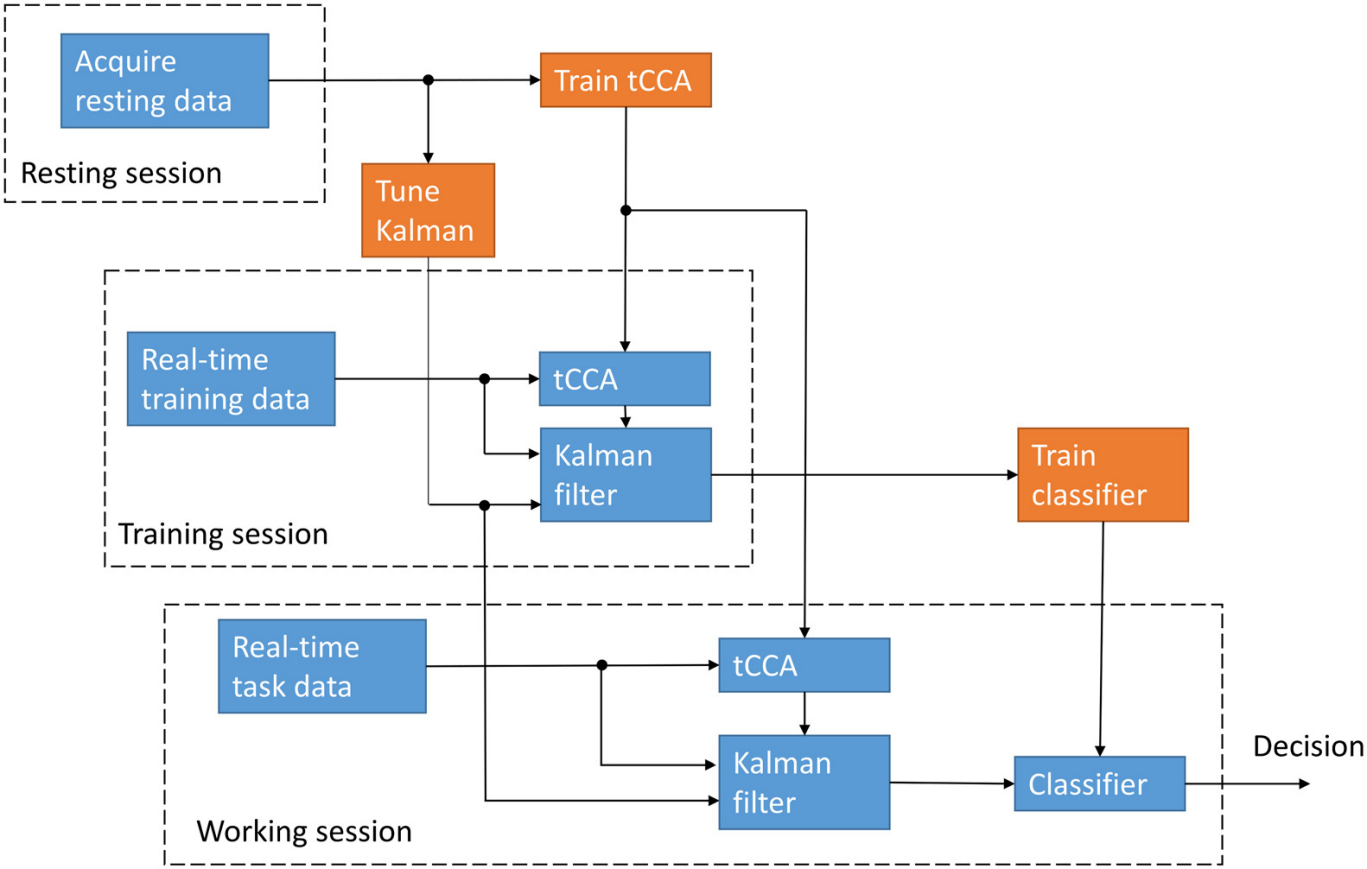
BCI paradigm for our multimodal online pipeline with three separate sequential sessions. Resting and training sessions are run first to train the BCI. Blocks in blue represent operations in real-time, and orange blocks are operations done in postprocessing.

Our multimodal online fNIRS BCI pipeline is shown in Fig. 1. It consists of three separate data acquisition sessions: resting, training, and working. During the resting session, a short (5 min) baseline fNIRS dataset and auxiliaries (in our case, short separation and accelerometer) are acquired. The resting data are used to train the tCCA filter and tune the Kalman filter. During the training session, the user is exposed to different stimuli and the generated fNIRS data are regressed in real time with the tuned Kalman filter and tCCA regressors. The resulting brain activation data are used to train a classifier (for example, with cross-validation). After these two sessions, the BCI is trained and ready to process the neural data and produce decisions in real-time. The fNIRS system cap should remain in the same position between sessions to better preserve the training and tuning statistics.

### 2.1 Prerecorded Subject Measurements

We use two different prerecorded datasets to test and demonstrate our algorithms. Dataset 1 consisted of fNIRS resting data measured from the visual cortex, and it was used to test and validate tuning strategies for the Kalman filter by augmenting it with synthetic HRFs. Dataset 2 was acquired from the motor cortex while subjects performed one of four different tasks (left and right overt and covert finger tapping). Dataset 2 also includes resting data for each subject. Dataset 2 was used to demonstrate real-time regression for a classification task.

Dataset 1 consisted of resting state time series for 14 subjects and was acquired with a TechEn CW6 system (NIRSOptix, Milford, Massachusetts). This system was used with 25 long separation channels (29.1 mm), 2 short separation channels (8 mm) as well as accelerometer, PPG, RESP, and BP data. This dataset is described in more detail at Ref. 30 and can be accessed at Ref. 31. The measurements were performed on the visual cortex. Each of the resting state time series lasts between 280 and 300 s, during which the participants were asked to limit their movement and no stimuli were presented (subjects watched a blank computer screen). The time series of each subject was split in two halves. One of the halves was used to train the tCCA weights and tune the Kalman filter (training set), and the other was augmented with synthetic HRFs to test the performance of the tuned filter (validation set).

**Fig. 2:**
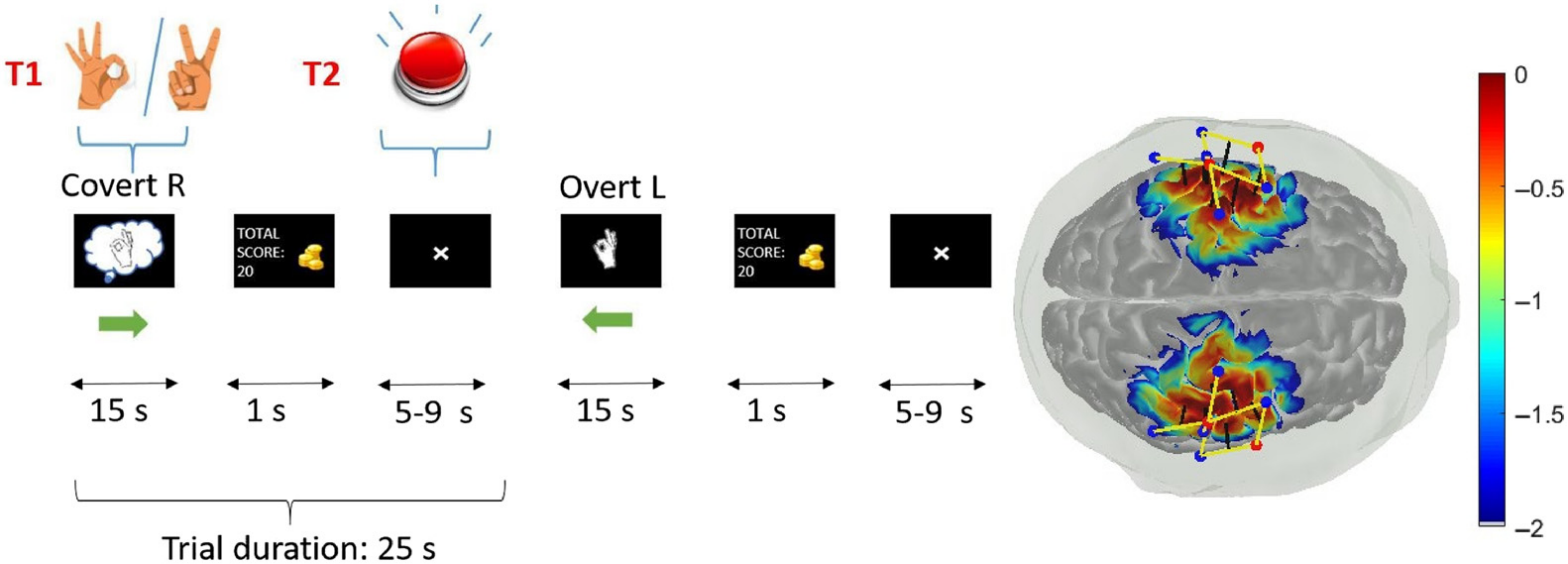
Cartoon of the task for the pre-acquired finger tapping dataset. The user was instructed to perform either covert or overt finger tapping with their left or their right hand for 15 s, which was followed by a rest period of 5 to 9 s. Covert action was indicated by an “ok” hand sign inside a “thought balloon,” while the “ok” sign with no thought balloon indicated overt action. Occasionally, a red button or “victory” hand sign would appear, which prompted the user to press a foot pedal. After the rest period, another condition would be presented and this process would repeat for a total of around 50 min. On the right, a diagram of the probe locations on the head (red dots are sources and blue dots are detectors) and their projection to the motor cortex, as well as the relative brain-sensitivity profile (indicated by a colorbar in logarithmic scale) are displayed.

For dataset 2, we collected fNIRS measurements from another set of 10 subjects using a custom built fNIRS system with silicon photomultipliers (SiPM) as detectors, operating at 100 samples per second. Measurements were collected with 12 long separation channels (i.e., 30 mm source–detector separations) measured at two wavelengths each (730 and 850 nm). The optodes were placed on the premotor cortex, with six of the channels on the right side of the head and the other six on the left side (see Fig. 2). In addition to the long separation fNIRS signals, we recorded a series of auxiliary measurements to aid in the regression: two short separation fNIRS channels (8 mm separation, one channel on each hemisphere) and three-axis accelerometer (x-y-z) signals. Both datasets were acquired in accordance with the regulations of the institutional review board of Boston University. The two datasets are briefly summarized in Table 1.

**Table 1:** Summary of the prerecorded datasets used in this work.

	Dataset 1	Dataset 2
Type	Resting data only	Left versus right finger tapping plus resting data
Number of subjects	14	10
Acquired on	Visual cortex	Motor cortex
Used for	Testing Kalman tuning strategies with synthetic HRF	Demonstrating online processing

In dataset 2, there are two time series for each subject. The first one is the “resting data,” which is a short duration measurement lasting 5 min during which the subjects are instructed to remain as motionless as possible, and no stimuli are presented to the subject (they observe a fixation point on the screen). The resting data of dataset 2 is used to train the classifier and the Kalman filter for online regression and classification. The second time series (the “working data”) was around one hour long for each subject. During its acquisition, the subjects were looking at a computer screen, which gave them instructions to perform one of four different tasks for 15 s. The four tasks were finger tapping with their left hand, finger tapping with their right hand, perform mental imagery of finger tapping with their left hand, or perform mental imagery of finger tapping with their right hand. The former two types of instructions are called “overt” and the latter two types are called “covert.” Between each stimulus, there was a random rest period between 5 and 9 s long. The four conditions of interest were repeated 27 to 30 times each. The task also included a mock feedback system, plus random catch trails (indicated by a red button or a “victory” hand gesture) for which the user had to press a foot pedal. There were also occasional random appearances of a red button during which the user also had to press a foot pedal. The catch trials and the red buttons, along with a scoring system, were meant to increase subject engagement. A cartoon of this process as well as the probe location and brain sensitivity profile (as indicated by a logarithmic color bar) are shown in Fig. 2.

### 2.2 Linear Model of the fNIRS Signal

The fNIRS signal is modeled as the following linear superposition:

$$y_{\mathrm{fNIRS}}=y_{\mathrm{HRF}}+y_{\text{drift}}+y_{\text{physio}}+\epsilon ,$$ (1)

where $y_{\mathrm{fNIRS}}$ is the measured fNIRS signals converted either to the hemoglobin concentration space using the MBLL or in optical density space.^32^ $y_{\mathrm{HRF}}$ is the change of the signal arising from the presence of stimuli activating the brain. $y_{\text{drift}}$ accounts for signal drift that can arise from the instrument or slow physiological changes in the subject. $y_{\text{physio}}$ is added to explicitly model confounding nuisance signals arising from nonbrain-related systemic physiology and artifacts, such as scalp hemodynamic fluctuations and motion artifacts. The ϵ term is the residual of the model fit to the data and is assumed to be a mean zero Gaussian distribution. The objective of the regression analysis is to find an estimate of these terms and thus separate the signal of interest $y_{\mathrm{HRF}}$ from the other terms.

We choose to model the HRF as a series of Gaussians, as this model does not make assumptions about the shape of the HRF or the timing of its peak.^33^^,^^34^ Thus, the HRF as a function of time is modeled as

$$y_{\mathrm{HRF}}(t)=\overset{40}{\underset{i=1}{\sum }}\beta_{i}e^{-\frac{1}{2}{\left(\frac{t-(i-1)\tau }{\sigma }\right)}^{2}}.$$ (2)

The parameters σ and τ are both 0.5 s, a set of values common in the literature (smaller values require more regression coefficients, whereas larger values reduce how fast the HRF can change). t=0 corresponds to the stimulus onset. The $\beta_{i}$ coefficients are the unknown amplitude of each Gaussian; finding them is the objective of the regression as they determine the shape and timing of the HRF. This model assumes the HRF goes to zero ∼20  s after the stimulus onset due to the duration of the trials.

For our state space model, the drift term $y_{\text{drift}}$ is defined as an unknown single constant γ. This is different from the usual practice of the GLM to model the drift as a third-order polynomial.^25^^,^^35^ The reason for this is that the Kalman filter algorithm allows γ to change over time, and thus we do not need to model gamma as a specific order polynomial.

The confounding external nuisance signals $y_{\text{physio}}$ are usually modeled simply as a weighted linear superposition of the auxiliary time series. However, for this work, we modeled them as composite, optimally delayed multivariate regressors, which we will describe in the following section.

### 2.3 Temporally Embedded Canonical Correlation Analysis

The confounding external nuisance signals are modeled as weighted regressors generated by temporally embedded canonical correlation analysis (tCCA) of fNIRS signals from long channels and multivariate auxiliary input signals (here: short fNIRS channels and accelerometers)^21^^,^^36^

yphysio(t)=∑jJCCAαjs^jCCA(t), (3)

where s^jCCA(t) are the latent components calculated from the auxiliary signals using tCCA. The $\alpha_{j}$ regression coefficients determine the contribution to the fNIRS signal from each latent component and are to be found with the Kalman filter. The s^jCCA(t) regressors are calculated from a linear combination of the auxiliary channels and D time-shifted copies, such that the regressors are maximally correlated to the noninstantaneous fNIRS time series in tCCA space. This way, the resulting regressors are optimized to remove the physiology as measured by the auxiliaries with the appropriate delay. Regressors are ranked by their correlation to the linearly mixed fNIRS channels in CCA space (the constant j is their rank). For this work, we used the two highest ranked tCCA regressors obtained using the accelerometer and short separation channels as nuisance signals. We choose only the top two to avoid overfitting and to make sure that the regressors had a correlation higher than 0.3 for all subjects. The temporal embedding parameters were chosen according to the guidelines for optimization by von Lühmann et al.^21^ as 0.08 s for the step size and a total time lag of 3 s (for a total of 37 time-embedded copies). We calculated the tCCA model with a custom-built Matlab function called rTCCA (with parameters: default flags, param.tau = 8, param.NumOfEmb = 37), which can be found on the GitHub repository available at: https://github.com/avolu/tCCA-GLM. This function uses regularized tCCA to reduce overfitting. The tCCA mixing model was estimated from resting data for each subject, and the resulting model was then used to calculate the regressors for the online Kalman regression, with the assumption that delays change little for the duration of the experiment.

### 2.4 State-Space Model for Kalman fNIRS Regression

We implemented a discrete-time Kalman filter state-space model as described on Gagnon et al.:^25^

x[n+1]=x[n]+w[n]y[n]=C[n]x[n]+v[n]. (4)

Variable names in bold represent vectors and matrices, whereas regular fonts represent scalars. Here, y[n] represents the n’th sample of the fNIRS measurement. x[n] is the state (column) vector containing the regression coefficients $\beta_{i}$, γ, and $\alpha_{i}$ (in that order). In our model, the state vector had a total of Nc=43 elements (40 coefficients for the Gaussian components of the HRF to cover 20 s after the stimulus onset, one for the drift term and two for the tCCA regressors). C[n] is a row vector containing the n’th sample of the regressors; the first 40 elements are the n’th samples of Gaussian elements of the HRF [Eq. (2), sampled to the sampling frequency of the fNIRS data], the 41th element is a constant (1) and elements 42 and 43 are the n’th sample of the first and second tCCA regressors, respectively. The product C[n]x[n] represents the linear mixing model shown in Eq. (1) for the n’th sample. v[n] is the measurement noise in the fNIRS signal. The term w[n] is called the process noise and describes how the regression coefficients drift over time. Both w[n] and v[n] arise from a stochastic process assumed to have zero mean and covariance Q and R, respectively (the former is an Nc-by-Nc matrix, whereas the latter is a scalar). w[n] and v[n] are assumed to be white and uncorrelated to each other. For our model, we assume that Q and R are time independent. This model treats each fNIRS channel independently and does not consider interchannel correlations. We assume each fNIRS channel has its own set of parameters Q and R, as well as a state vector independent from the state vector of the other channels. This essentially means that each channel requires two Kalman filters running in parallel (one for each wavelength/chromophore). The Kalman filter recovers the best estimation of the regression coefficients (x[n] the state of the system, and thus, the shape of the HRF) from the noisy measurements y[n] given some prior knowledge of the statistical properties of the system. More details about the Kalman estimation procedure can be found in the appendix of this work.

### 2.5 Kalman Filter Algorithm and Tuning

We propose a strategy for tuning the Kalman filter using resting data to optimize its performance when regressing fNIRS data online with tCCA regressors. Tuning the Kalman filter consists of (1) adequate estimation of the process and measurement noise covariances Q and R and (2) finding priors for the state of the Kalman filter. There are two priors to specify in the Kalman filter: an estimation of the state of the system x (the regression coefficients) at the start of the experiment and an estimation of the error covariance matrix P at the start of the experiment (for more details on the definition of these variables and the tuning, see Appendix). First step in the workflow for tuning the filter for a subject and experimental paradigm is to acquire a resting state time series for the subject. Then, the resting data can be used to estimate the noise covariances Q and R, as well as some elements of the error covariance matrix, P. Note that the components of Q corresponding to the HRF were set as zero for this work, implying that the HRF is expected to be consistent between trials (this is discussed further in Appendix). The state of the system can be initialized as a zero vector, which has the advantages of simplicity and reduced bias toward a known solution, but the disadvantage of a slower convergence.

We used dataset 1 to evaluate the performance of our tuning strategies. For this, we split each of the 14 resting time series into two halves: one was kept unmodified to use for tuning the filter (training set) and the other half was augmented with known synthetic HRFs (validation set). Only approximately half of the channels in the validation set were augmented (chosen with a probability of 50%). This procedure is similar to the one used by von Lühmann et al.^21^ The synthetic HRFs have an amplitude typical of HRFs observed in real experiments (on the order of 1  μM) and each of the augmented time series had up to eight repetitions of this synthetic HRF appearing at ∼20  s intervals with a ±3  s random jitter. The training set was used to estimate the noise covariances and the error covariance matrix. The Appendix provides more details on how we processed the resting state data to achieve this. The validation dataset was regressed using the tuned filter to obtain the estimated HRF as a function of time. We compare this temporal evolution of the estimated HRF with the ground truth HRF to calculate the evolution of the RMSE as a function of time. We normalize the RMSE evolution to the final RMSE obtained from performing GLM regression to the validation data. This reference GLM regression was calculated with the functions included in the Homer2 toolbox,^37^ specifically the function hmrDeconvHRF_DriftSS, which allowed us to pass the tCCA regressors to the GLM (the consecutive Gaussian with a width and a separation of 0.5 s as in the Kalman model, up to 20 s after the stimulus, and order 3 for the drift term). This enabled a performance comparison of both techniques, to see if or when the Kalman filter performance improves upon the performance of the GLM. Finally, we calculate the mean and standard deviation of the HRF RMSE evolution across all subjects. For comparison purposes, we also performed this process with an “untuned” Kalman filter. For the “untuned” filter, the parameters were chosen based on our previous experience with offline Kalman filters developed from using typical values reported in the literature as a starting point.^24^^,^^25^^,^^38^ The “untuned” parameters were: P as an identity matrix scaled by 1e-9, R=1e−8 for all channels, Q an identity matrix scaled by 1e-17, and the starting estimation of the system state x[0] equals to a zero vector. These values are assuming the fNIRS data are expressed in concentration (M). This way, we can compare the performance of our proposed tuning to a set of arbitrary values previously shown to perform well but not necessarily optimally.

The validated tuning strategies were then implemented as a part of a real-time prerecorded pipeline used to regress the finger tapping data with the purpose of classifying the evoked hemodynamic responses to the brain activation. The implementation of the Kalman filter in this case is slightly different as it is a single stimulus regression. For this reason, for the pseudo-online implementation of the Kalman filter, we reset the HRF components of the state of the Kalman filter at each stimulus onset.

### 2.6 Real-Time Prerecorded Pipeline

We developed a real-time prerecorded pipeline to regress the fNIRS data in dataset 2 in real-time and investigate the effects of regression in the classification performance as left versus right finger tapping. The pipeline applies the Kalman filter to the time series in real-time (or faster) to demonstrate the feasibility of multimodal online regression, and features are extracted by calculating the moving average of each regressed channel in windows of 3 s. These features are then used with 10-fold cross-validation (with strict split of training/test/validation sets) to train and test the rLDA classifier^39^ to predict the binary (left versus right) classification performance (segregated by overt and covert cases). Once trained, the classifier can assign a class to a set of features by calculating a simple dot product.

**Fig. 3:**
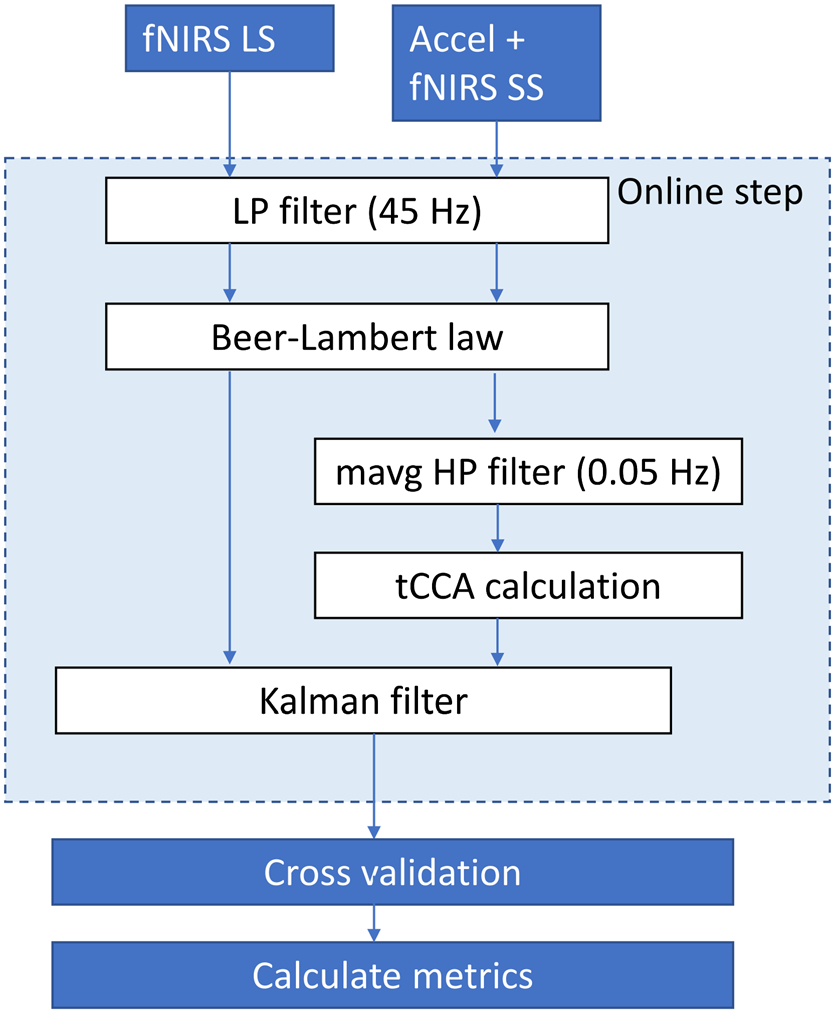
Block diagram of the real-time prerecorded processing pipeline to calculate the Kalman filter regression of the fNIRS data (online part, encased in light blue rectangle) and then estimate the classification accuracy of the regressed data (offline part, immediately after Kalman filter).

The processing pipeline is shown in Fig. 3. As a preprocessing step, the corresponding resting state data for the subject were used (1) to obtain the linear mixing model necessary for creating tCCA filters and (2) to tune the Kalman filter. The tuning was performed as described in Appendix (with the caveat that some elements of the parameter P are initialized with an empirical arbitrary value optimized from synthetic data, Appendix 6.7). Then, the working data are read from the file and streamed through a TCP/IP network using Labstreaming layer (LSL is available in a GitHub repository: https://github.com/sccn/labstreaminglayer). This is done with the purpose of simulating a multimodal experiment in which the data are being collected by several instruments and collected by another computer. The data are streamed at an accelerated rate compared with its real acquisition (up to four times the original) to both accelerate the reacquisition and to show that the Kalman filter can perform the processing in real-time even with an increased sampling rate. The streamed data are received by another script to perform the real-time regression (the blocks marked as “online step” in Fig. 3). The data are first low pass filtered at 45 Hz (Butterworth order 5); this filter exists mainly to reduce any possible contribution from the AC power while still keeping the fast components of the signals during the tCCA analysis. The fNIRS data are then converted to chromophore concentration changes using the MBLL (for the single wavelength analysis, the data are only converted to difference of optical density). Simultaneously, the auxiliary channels are high pass filtered at 0.05 Hz (to remove their DC component) and then used to calculate the tCCA regressors with the tCCA filter calculated from the resting data. After that, both the fNIRS concentration data and tCCA data packages are sent to the tuned Kalman filter for regression. As this is a classification problem, the Kalman filter is provided with the timing of the stimuli but not their class. The state of the Kalman filter for the HRF is reset at each stimulus onset to prevent prior HRF estimates from biasing the results. The output of the Kalman filter is a real-time estimation of the HRF y^HRF[n]. This HRF time series is streamed to disk as it is calculated.

After completing the reacquisition and regression for the subject, the file with the HRF time series is read (offline) by another script to train and evaluate the classification performance using cross-validation. The stimulus timing and class information is passed along with the time series to the offline classifier script. All 24 HRF channels (12 HbO and 12 HbR) are passed to the cross-validation block. The channels with SNR<2  dB are rejected. The HRF channels are low pass filtered at 0.1 Hz with a third-order Butterworth filter. Next, the time series are divided into epochs according to the stimulus type. The epochs are segregated into overt and covert responses. A baseline removal is performed on each epoch by subtracting the mean value of the signal in the interval from −2 to 0 s (prestimulus). Classification performance is evaluated using 10-fold cross-validation with a binary (left versus right) rLDA classifiers with automatic shrinkage parameter selection.^40^ We used one classifier for the covert responses and a separate one for the overt responses. The classifier evaluates the validation data by performing a time average of the signal in windows of 3 s of length. The first 3 s after the stimulus onset for each epoch are averaged to get as many features as channels received by the classifier (up to 24). These features are passed through the previously trained rLDA classifier for the appropriate condition (overt or covert) and a decision is made (left versus right). To investigate classification performance across time after onset, we repeat this approach with a rolling 3 s window, iteratively shifted by 0.2 s, across the whole 20 s epoch. This provides statistics for classification performance across subjects and trials as a function of time after the stimulus onset. We used those to estimate the classification accuracy into left versus right. The classification performance for each subject as a function of time after the stimulus is saved in a file. Then, the whole process (reacquisition, online regression, and cross validation) is repeated for all 10 subjects in the dataset, and average classification performance is calculated for all subjects. We also performed the process described in Fig. 3 with the Kalman filter turned off, so we could compare the classification performance and spectral characteristics with and without Kalman filter regression.

In addition, to understand the spectral effect of the Kalman filter on the data, we calculated the power spectral density (PSD) of the data before and after regression. For this, we used Welch’s method [“pwelch” function with default parameters in MATLAB 2020a (Mathworks, Natick, Massachusetts)].

All the processing was performed in MATLAB using custom made scripts as well as external toolboxes Homer2, BBCI,^41^ and LSL. The datasets can be accessed at Refs. 31 and 42. Custom-made functions for this work and examples on their usage can be downloaded from GitHub repository: https://github.com/BUNPC/KalmanTuning.

### 2.7 Contrast-to-Noise Ratio

We calculated the average contrast-to-noise ratio for the different HRF classes in dataset 2. This allows us to quantify and compare the amount of noise obscuring the HRF before and after the Kalman regression. For this, we first calculate the CNR for each epoch as

$$\mathrm{CNR}=\frac{\left|{\left\langle \mathrm{HRF}(t)\right\rangle }_{5s}^{10s}-\text{mean}(\text{baseline})\right|}{\sqrt{\mathrm{var}(\mathrm{HRF})+\mathrm{var}(\text{Baseline})}},$$ (5)

here HRF(t) is the time period after the stimulus onset. We calculate its mean from 5 to 10 s and subtract the mean of the baseline (−2 to 0 s) for the numerator. The denominator is simply the variance of the HRF plus the variance of the baseline. We calculated this metric for all epochs and all channels of all subjects and then calculated averages of the CNR by stimulus type (overt/covert left/right) and chromophore (or wavelength in OD space). The average CNRs were calculated across all channels and subjects. We also performed this calculation for the case with no Kalman regression to estimate the mean improvement in CNR.

### 2.8 Single Wavelength Classification

The processing flow to analyze single wavelength classification was similar to the one used for regular classification in the concentration space, with only two differences. The first one is that the MBLL block was modified to stop after transforming the fNIRS signals to optical density (a required intermediate step to converting them to hemoglobin concentration space), and thus, to return the data in OD space instead of concentration space. The second modification was changing what channels are sent to the cross-validation algorithm. For the concentration space, we used values from all channels as features for the classifier (12 HbO and 12 HbR channels); for the single wavelength classification in the optical density space, we only used either the 12 channels for the red wavelength or the 12 channels for the infrared wavelength so we can compare the performance at each wavelength. We also performed the calculation in the OD space when sending the channels of the two wavelengths to compare the performance in concentration space versus OD space. The Kalman tuning in the OD space is performed the same way as in the concentration space, with the difference that the tuning and tCCA algorithms receive the resting state data in optical density space instead.

## 3 Results

### 3.1 Kalman Tuning Strategies on Synthetic Data

**Fig. 4:**
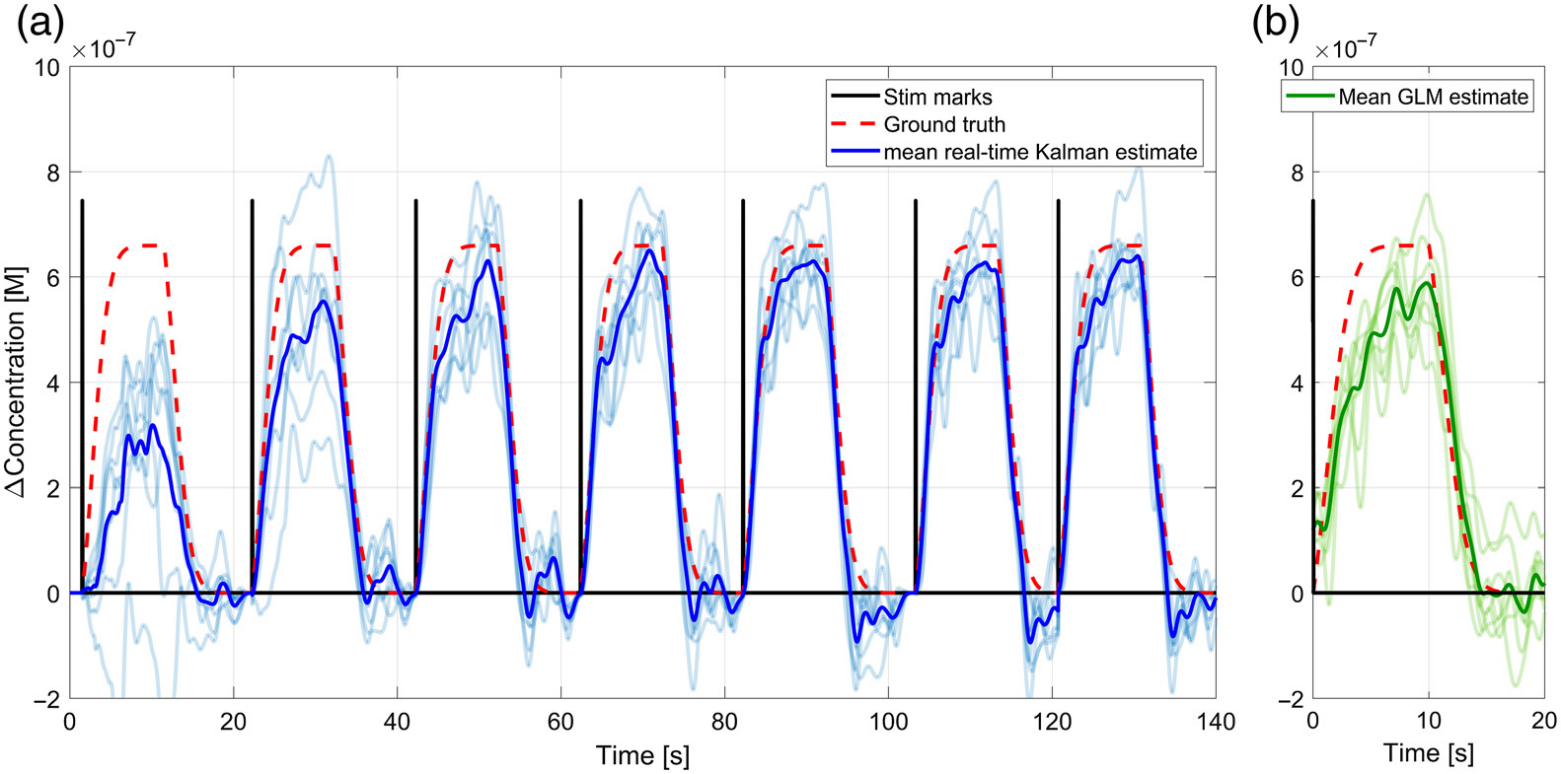
(a) Evolution of the estimated HRF by the tuned Kalman filter as a function of time and (b) GLM esimate of the HRF for the complete time series. This plot shows the estimation of the HRF for the channels augmented with a synthetic HRF for one of the subjects (visual cortex dataset). The solid lines in the figures are the mean for all channels for the real-time Kalman estimation (blue) and the mean for all channels /for the *a posteriori* estimation with the GLM (green). The semi transparent lines are the individual channel estimations for the Kalman (blue) and for the GLM (green). The red dashed curve is the ground truth, and the vertical black lines represent the stimulus onsets.

Figure 4(a) shows an example of how the estimation of the HRF evolves with time with the tuned Kalman filter. The figure shows the HbO regression for the resting state visual cortex fNIRS data augmented with a synthetic HRF for one subject. The vertical black lines represent the stimulus onsets. The dashed line is the ground truth (the synthetic HRF added to the data). The bold solid line is the mean real-time Kalman estimate across channels augmented with an HRF (channel with no HRF added are not shown). In Fig. 4(b), we show the estimate of the HRF using the GLM regression for the complete time series. The faint lines on each panel represent the individual channels.

**Fig. 5:**
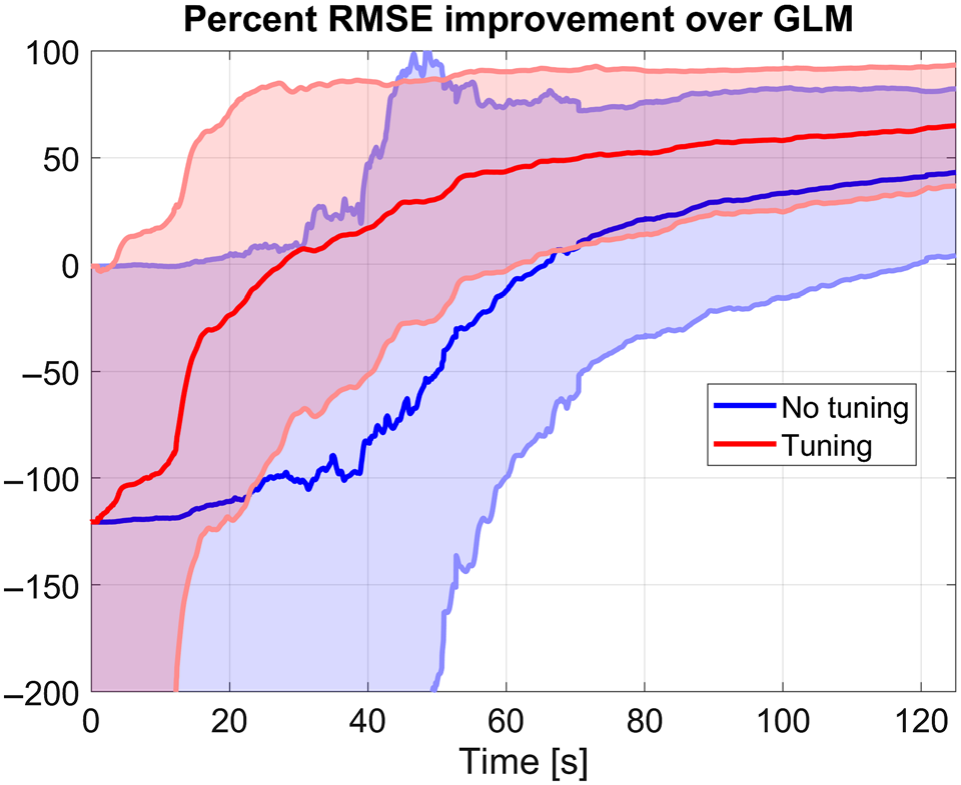
RMSE evolution of the Kalman regression. Mean (solid lines) and standard deviation (shaded areas) of RMSE across subjects and channels. The red curves represent the relative RMSE for the tuned Kalman (with respect to the GLM RMSE), and the blue curves are results from arbitrary empirical tuning.

Figure 5 shows the temporal evolution of the RMSE for the dataset augmented with synthetic HRFs when regressed with the Kalman filter and tCCA. The RMSEs are expressed as a percentage of the RMSE obtained from regressing the complete data series with the GLM ($\frac{{\mathrm{RMSE}}_{\mathrm{GLM}}-{\mathrm{RMSE}}_{\text{Kalman}}}{{\mathrm{RMSE}}_{\mathrm{GLM}}}x100\%$). Thus, a zero on the y-axis indicates equal RMSE performance as the GLM, a 60% on the y-axis indicates that the Kalman filter RMSE is 0.4 times that of the GLM and so on. Note that the GLM is calculated for the complete time series, whereas the Kalman filter is evaluated sample-by-sample. The state vector is initialized as zeros, thus the initial RMSE for the Kalman filter starts at −100%, but it improves as time passes (and more repetitions of the HRF appear on the time series). For this particular tuning, we used an estimation of the error covariance matrix from a previous run of the regressor with suboptimal parameters (see Appendix 6.7). We also present the mean and standard deviation of the relative RMSE when arbitrary empirical values of the Kalman parameters are used (labeled as “untuned”) to compare how tuning compares to the GLM approach.

### 3.2 HRF Kalman Regression of Overt and Covert Finger Tapping Data

**Fig. 6:**
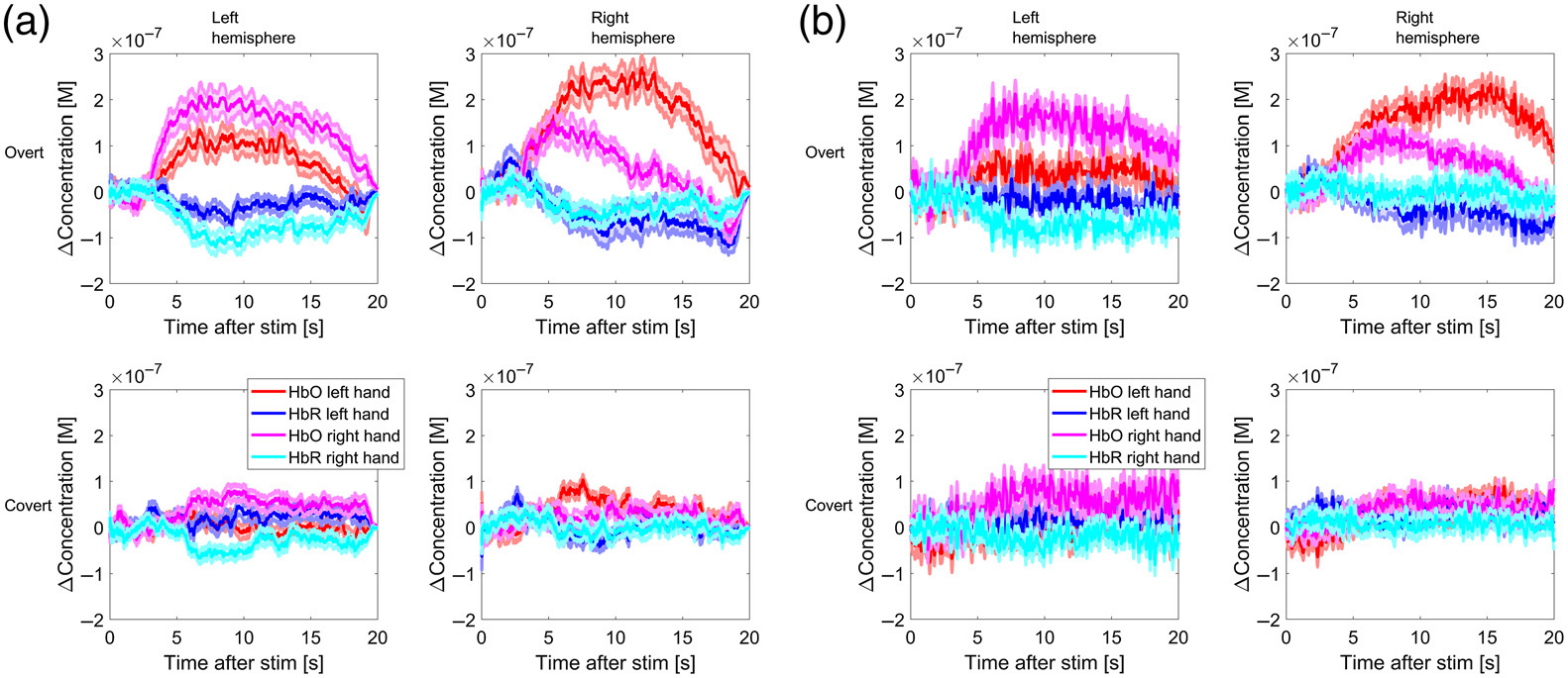
(a) Block averages of HRFs after real-time Kalman regression for the finger tapping dataset and (b) block average of raw time series for comparison. The averages were calculated across trials, subjects, and channels, but segregated by stimulus type (overt/covert left/right) and brain hemisphere. The HRFs for overt task are on the top row and for covert task on the bottom row. Each of the four different colors of curves represents the response to a different type of stimulus. The shaded areas are the standard error (60 curves were averaged to produce these plots).

Figure 6 shows the mean HRFs obtained from the real-time single stimulus Kalman regression of the finger tapping dataset (a, left) and the block averages for the raw (low pass filtered at 1 Hz) data (b, right). The HRFs in the figure are averaged across subjects and channels but segregated by stimulus type, chromophore, and brain hemisphere.

**Fig. 7:**
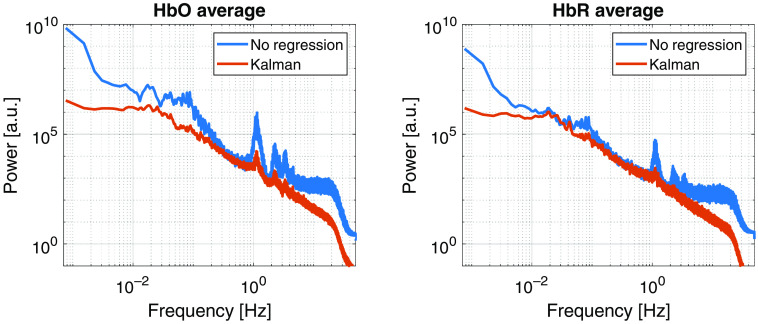
PSDs for the time series of one of the 10 subjects of the working set for the finger tapping data before (blue) and after (red) Kalman HRF regression. The spectra on the left are for HbO and the ones on the right are for HbR.

Figure 7 shows the average (across channels) HbO and HbR PSD for one of the 10 subjects of the finger tapping dataset. The blue curves are the PSD for the time series with no regression, i.e., after the data are converted to chromophore space and 45 Hz low pass filtered. The red curves show the PSD after the data were filtered with the tuned Kalman filter.

Table 2 reports the average contrast-to-noise ratios obtained for the different conditions and stimuli, for both nonregressed (Kalman filter off) and Kalman regressed, as well as the regression improvement (the ratio between the regressed and nonregressed CNR).

**Table 2:** Average CNRs for the different HRFs before and after Kalman regression, as well as improvement with regression (ratio of regressed CNR to non-regressed CNR).

	Overt no regression	Overt Kalman regression	Overt improvement	Covert no regression	Covert Kalman regression	Covert improvement
HbO	1.20	4.73	3.94	0.343	1.34	3.91
HbR	−0.577	2.51	4.35	0.310	1.32	4.26
OD730 nm	0.335	2.00	5.97	0.213	1.28	6.00
OD850nm	0.842	4.67	5.55	0.330	1.88	5.70

### 3.3 Classification Performance (Concentration Space)

**Fig. 8:**
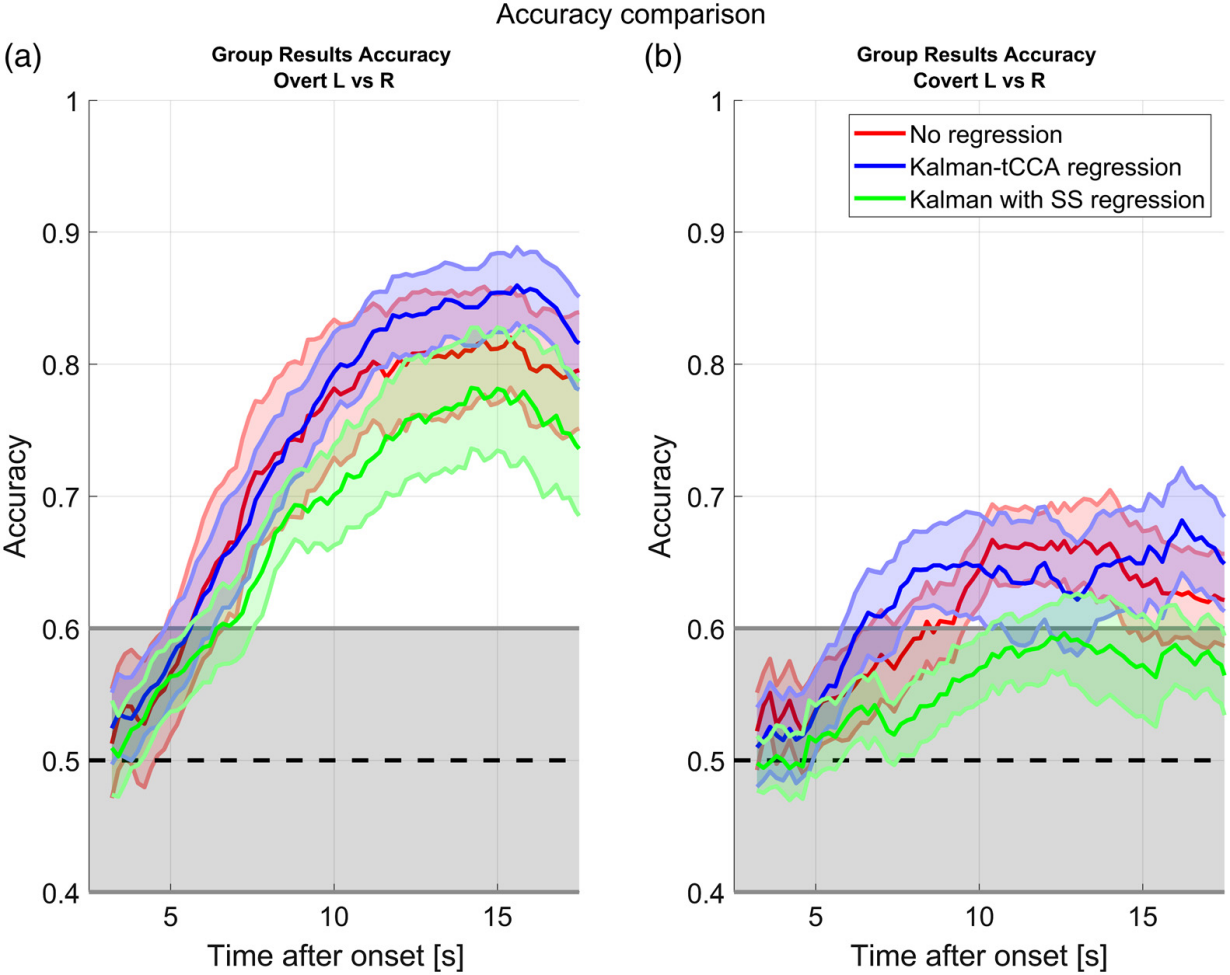
Mean (solid) and standard error (shaded) classification accuracy for the finger-tapping data estimated with cross-validation and an LDA classifier. The blue curves are for the data after HRF Kalman-tCCA regression. The red curves are for the time series with no regression. The green curve is for Kalman regression with short separation channels but no tCCA. (a) The overt responses and (b) the covert responses. The area shaded in gray represents the region where we cannot discard the null hypothesis that the classification accuracy is better than random guessing. The statistics were calculated across subjects (10 subjects) and trials (27 to 30 trials per condition). The classifier features were the means of the channels over 3-s windows. The curves show how the classification accuracy progresses after the stimulus onset.

The classification performance for our rLDA classifier is summarized in Fig. 8 for the overt and covert cases. The solid blue line is the mean classification accuracy across 10 subjects when the classifier is fed by the signal after Kalman HRF regression. The shaded region represents the bound of the standard error estimated across all the subjects. The red curves are for the case where the signal was not regressed and are shown for comparison. The green curves are also for reference and represent Kalman-regressed data where the physiology has been modeled simply as the short separation channels (with no tCCA). The left panel is for the overt stimuli, whereas the right panel is for the covert stimuli. The x-axis represents the classification accuracy obtained for a 3-s window. For example, the data at 5 s represent the classification obtained by averaging the data from 2 to 5 s. The black dashed line is the 50% accuracy line; any accuracy lower than that is worse than random chance level. The gray shaded region represents the bounds of the region where we cannot reject the null hypothesis that the classification accuracy is worse than random guessing (calculated with the binomial distribution and 27 trials and α=0.05). By presenting the data like this, we can estimate how much time after the stimulus onset we need to wait before obtaining a better performance than random guessing. The plots also show, on average, when the maximum classification accuracy for fNIRS signal happens. A paired sample student t-test did not find a statistically significant difference between the accuracy curves for the Kalman-tCCA regression versus the no-regression case at their peak difference (p=0.056 and p=0.17 for the overt and covert cases, respectively, at 16 s).

### 3.4 Classification Performance (Single Wavelength)

**Fig. 9:**
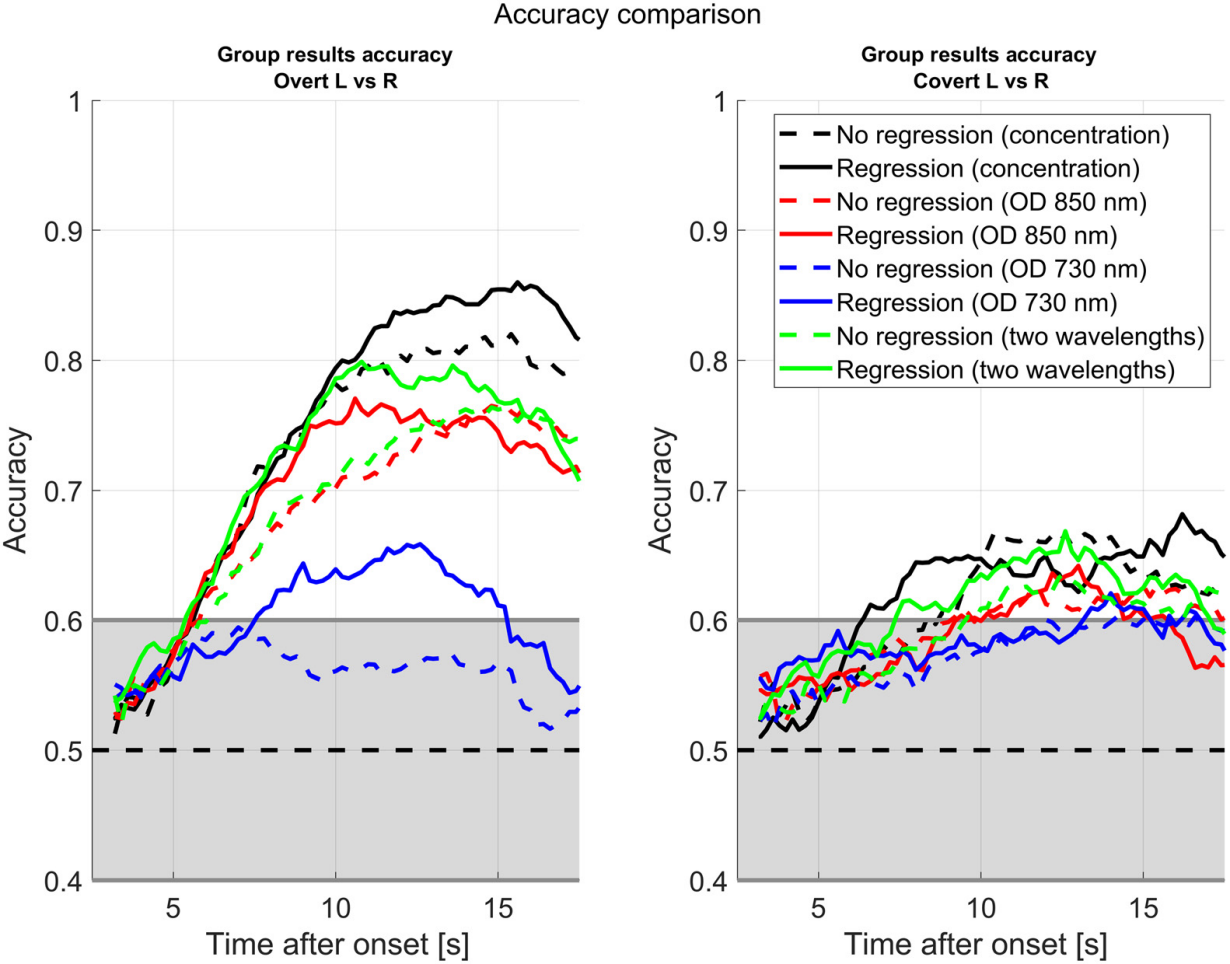
Mean classification accuracy for single wavelength fNIRS data in optical density space. Solid lines are for data regressed with the Kalman filter, and dashed lines are for data with no regression. The black lines are for reference and represent the classification accuracy for the data in the hemoglobin concentration space.

Figure 9 shows the results for the mean classification accuracy for single wavelength classification in the optical density space (as it is not possible to convert to the concentration space using a single wavelength). Solid lines are for the mean classification performance after regression, whereas dashed lines are for the mean accuracy with no regression. The red curve is for the 850 nm and the blue for the 730 nm. This was achieved by passing the channels of only the respective wavelength to the classifier. The green curves are the classification accuracy when the channels of both wavelengths are sent to the classifier in optical density units. The black curves are for reference and show the mean classification accuracy when the classifier is fed by all concentration space channels (the same means as presented in Fig. 8). The t-test resulted in a statistically significant difference in accuracy for the OD data at 730 nm when comparing the Kalman-regressed data with the nonregressed data (p=0.03 for the overt case, for the covert p=0.15 case).

## 4 Discussion

### 4.1 Kalman Tuning

The main barrier to entry in using the Kalman filter is the necessity to properly tune it to get optimal performance. Kalman tuning is far from a solved problem, and various strategies have been proposed over the years, either to estimate the tuning parameters offline from the data or to calculate dynamic estimations.^43^^–^^46^ Furthermore, the fNIRS Kalman literature obviates the tuning process as authors either select arbitrary values or try different parameters until they get a desirable result.^24^^,^^25^^,^^38^ In this paper, we developed a new strategy to tune the Kalman filter using resting data, specifically in a Kalman filter formulation using tCCA regressors, which have not been used in a Kalman filter framework before.

Our tuning strategies, on average, produce better final performance (60% smaller RMSE) than the GLM (see Fig. 5). We compared to the GLM as it is the gold standard for fNIRS regression, because it is difficult to compare to other Kalman models without first developing appropriate tuning for those models. We show that no tuning, i.e., using arbitrary initial values based on literature and previous experiences, also eventually produce better results than the GLM. However, the tuned filter shows faster convergence, requiring less repetitions of the trial to outperform the GLM. This despite the large parameter search space, showing that extracting the Kalman tuning parameters from the resting data is a sound strategy. In addition, the variance of the RMSE for the tuned case is smaller than for the nontuned case, as better selected parameters produce less oscillations in the response while the filter converges to the optimal gain.

In Fig. 4, we can see that even with tuning, not all channels display a good estimate of the HRF after regression during the first stimulus, but they will tend to get better as more repetitions are observed [Fig. 4(a)]. Even then, the mean estimated HRF starts getting very close to the ground truth and outperforms the GLM after only a couple of repetitions of the stimulus, even though the GLM was employed on the whole time series, including all trials.

The main drawback of our tuning strategy is that it requires having a resting state dataset available for the specific subject, probe design, and fNIRS system. The acquisition of resting data adds complexity to the experiment and makes it longer, so ideally it needs to be minimized while being long enough to capture the statistical variations of the system. Wang et al.^47^ showed that 2.5 min was enough to capture those variation in resting data, which should be relatively easy to obtain before the classification task. We demonstrated that this duration provides good results with synthetic data, and our simulated working sessions use a 5 min resting dataset for tuning. It is also likely that some of the parameters of the Kalman filter are relatively constant between experiments and thus could be reused without recording a resting dataset. For example, we might expect the measurement noise R of a given channel to be relatively constant, or the error covariance matrix for the HRF components to be similar across subjects for a given experimental paradigm. However, for our visual cortex dataset (dataset 1), we found the measurement noise of each channel to have some variation between subjects (coefficient of variation close to 1 for most channels). This can be attributed to differences in coupling of the optodes to the scalp between subjects and to the effects of motion artifacts in the spectral band used to estimate the measurement noise. In addition, channels receiving a stronger signal will also have more optical shot noise. On the other hand, with the synthetic data we observed that the error covariance matrix P estimated from a suboptimal regression of a single subject can be used as the initialization of P for other subjects, and this yields better results than using a typical diagonal initialization of P. As this was tested with synthetic data in which the HRF is identical between subjects, the results might be different when using this approach for real HRFs (expected to have variability between subjects). Further investigation is needed to determine if reusing filter parameters for different subjects yields better results compared to choosing arbitrary values.

Prior studies employing the Kalman filter cited in this paper tend to ignore the covariances between regression coefficients and initialize P as a diagonal matrix under the premise that the filter will eventually converge to a better estimation. However, the regression coefficients for the Gaussian model of the HRF are correlated, as they tend to move together. We observed that starting with a diagonal matrix causes a flat time evolution of the RMSE at first (no improvement during the first one or two stimuli) followed by an improvement around the first 20 to 40 s of the “untuned” curve, Fig. 5. The final estimation of P returned by the Kalman filter displays off-diagonal elements for the elements of the HRF, with covariances for adjacent regression coefficients. This suggests that the filter first needs to find the covariances between coefficients before it starts converging to the solution and that the optimal initialization of P should account for the off-diagonal terms in for a single trial regression.

On the other hand, the strategies we used to estimate the error covariance P for the visual cortex dataset were not effective for the finger tapping dataset. The reason for this is that the optimal value of the HRF components of P depends on the expected HRF. However, in a classification problem, we cannot make assumptions about P without biasing the filter to a response with a specific shape. For this reason, for the classifier implementation, we initialized P as a scaled identity matrix, with the scale determined semiarbitrarily by choosing a value that minimized the RMSE for the synthetic data. This value is in the order of magnitude of the amplitude of typical HRFs observed with fNIRS.

When devising tuning strategies, we observed that choosing an appropriate measurement noise R is important, as overestimating it causes slow convergence while underestimating it causes large spikes in the output at the stimulus onset (overcorrections). As this parameter is relatively easy to calculate, it is highly recommended to do so for any implementation of the Kalman filter, even if other parameters are chosen arbitrarily.

One of the advantages of the Kalman filter is that, as regression coefficients in general are not assumed to be constant in time, it allows for nonlinear relationships in time between regressors and signal. However, this can produce overfitting. As the drift term is simply a scalar that changes over time, an unwise selection of Kalman parameters might make the term follow the fNIRS signal. For this reason, we need to be very conservative with the selection of both the *Qdrift* (which determines how fast we expect the drift to change at each time step) and the *Pdrift* (which estimates how far the initial estimation of the drift is from the actual value) elements. Fortunately, the estimation of those parameters from the resting data consistently gave good results, and thus this stresses the importance of having a strategy to select those parameters.

Our tCCA tuning uses the same tCCA filter, regressors, and Kalman parameters for all channels (except drift and measurement noise parameters, which are specific to each channel). It is plausible that the regressor mixing is different for different channels. For example, we could expect for channels spatially closer to the short separation measurements to have shorter time delays than those of channels farther away. Similarly, some regions of the head may be more affected by the hemodynamics of the scalp, and thus some channels could have higher weights. For that reason, in the future it might be worth exploring using different tCCA regressors for each channel or a group of neighboring channels. This would increase the processing and memory requirements for the Kalman filter, creating additional constraints for real-time regression. In addition, generating tCCA regressors for each channel is prone to overfitting, so the implementation should be done with great care to prevent this.

### 4.2 HRF Kalman Regression of the Finger Tapping Data

After developing our tuning strategies with the resting state visual cortex dataset augmented with synthetic HRFs, we used those strategies to tune a Kalman filter for real-time regression of prerecorded finger tapping data. Since this is a classification problem, the subject is reacting to different classes of stimuli, and this makes it necessary to reset the HRF components of the Kalman filter at each stimulus onset. Otherwise, the estimation of the HRF for the previous stimuli will bias the estimation of the HRF for the current stimulus. We allowed the other components of the Kalman state (those related to the drift and tCCA) to continue to evolve between stimuli, as we want all the prior data to inform the future estimates for these other components of the Kalman state.

Figure 6(a) (left) displays the average HRFs for each stimulus condition. As expected, the response on the left hemisphere is stronger when finger tapping with the right hand; the response was stronger on the right hemisphere when finger tapping with the left hand. The overt response is stronger than the covert response. The covert response is barely discernable from physiological fluctuations albeit some sign of contralaterality. These plots serve as a “sanity check” to show that the Kalman filter can produce reasonable single stimulus estimations of the HRF without making assumptions about its shape and without overfitting while at the same time being less noisy than filtered raw data [Fig. 6(b), right]. As a reminder, these HRFs are averaged across subjects and trials; it is possible that the response for some subjects is small or nonexistent, and thus the average response will have a smaller amplitude than the typical amplitude when a response does happen. In fact, we found that for some subjects it was not possible to observe the HRF, which resulted in low classification accuracy. This is likely due to the subjects either not performing the task at all or using inefficient mental strategies. In this sense, it has been proposed that BCI use is a skill (BCI literacy) that needs to be developed effectively to obtain good results.^48^

The regression was calculated at a speed four times faster than real time (at an effective rate of 400 samples per second) with no data loss. This shows that our implementation of the Kalman filter is computationally efficient enough to regress the data in real time for applications such as BCI. Whether real-time Kalman regression will work or not for a given experiment will depend on the number of channels to be processed, the number of regressors, the sampling rate of the data, and the specific computer hardware and software used. As a reminder, the dataset in this study has 24 long separation channels (12 HbO, 12 HbR), 2 tCCA regressors, and was originally acquired at 100 samples per second (but transmitted at four times the rate to test the processing speed). We did the processing on a regular desktop Windows computer (16 GB RAM, solid state drive and 3.6 GHz seventh generation i7 microprocessor). While fNIRS systems with a much larger channel count can challenge the real-time processing, it should be noted that in general commercial fNIRS systems tend to have much lower sampling frequencies (10 Hz or less), which reduces the necessary data processing rate.

From the PSDs of Fig. 7, we can observe that the Kalman tCCA regression produces a considerable reduction in the spectral power (around two orders of magnitude for HbO) of the cardiac pulse (observed as a peak around 1 Hz) for both the HbO and HbR signals while also reducing the power of the harmonics. In addition, the Kalman is producing a low-pass effect (as the HRF regression model is composed of low frequency components) and simultaneously a high-pass effect (as the offset of the signal is removed by the drift term). The regression seems to also have reduced the power of the Mayer waves component (around 0.1 Hz). As the only filter used before the Kalman regression was a 45 Hz low-pass filter, this provides evidence that the Kalman filter can selectively remove spectral components without removing the desired parts of the signal in the same frequency. However, the reduction of the cardiac pulse power is variable across subjects, sometimes producing a smaller reduction when the SNR of the resting data was lower.

### 4.3 Effect of Regression on Classification Accuracy

Our classification accuracy plots (Fig. 8) show that the fNIRS data can be used to determine if the hemodynamic response was produced by finger tapping with the left hand or by finger tapping with the right hand with a good degree of classification accuracy. This works both for actual motor performance and for motor imagery, though it is more effective for the former. One of the reasons that makes classification into left versus right possible is that the hemodynamic response is lateralized, as evidenced by the average HRFs (Fig. 6). The average HRFs also provide some insight on why the classification of covert responses is generally worse compared with that of the overt. The covert HRFs have on average a much smaller amplitude than the overt, to the point that the contralateral responses are on average dominated by confounding physiological fluctuations rather than the evoked hemodynamic responses in the brain. Furthermore, it has been shown that the strength of the covert response is highly dependent on the mental strategy used by the subject to perform the imaginary finger tapping.^49^ This is evidenced by the fact that the mean covert accuracy for some subjects reached 80%, whereas for other subjects it never left the random chance region. The other calculated metrics (such as specificity and sensitivity) follow almost identical temporal profiles as the accuracy, and thus we decided to omit them in this paper.

Multimodal Kalman regression seems to increase the classification accuracy during the 7 to 12 s time period after the stimulus when the signals are in the optical density space (overt case). Single wavelength classification seems to be much worse than the classification in the concentration space, but this difference gets smaller after regression, especially for the time interval between 5 and 10 s after the stimulus. However, at least in the chromophore space classification (Fig. 8), these differences failed to be statistically significant. We only observed a statistically significant change for the single wavelength 730 nm signal (and for classification with HbR channels only). This does not necessarily mean that filtering the signal is ineffective in increasing the classification accuracy, but it could simply mean the effect is not big enough for the statistical power of our tests. It is also very likely that some of the classification accuracy in the case with no regression is provided by the extracerebral signals, which the Kalman filter regression is meant to remove. For example, it has been shown that there are task-evoked hemodynamics changes in the skin,^12^ which the tCCA regression is meant to remove. It is also possible that the physical act of performing the finger tapping produces lateralized subtle changes in position and blood pressure that propagate to the fNIRS signals and are picked up by the classifier, artificially inflating the classification performance of the raw signals. This is suggested by the classification accuracy of the Kalman regression with short separation but no tCCA (Fig. 8, green curve); removing the systemic physiology but not the motion artifacts (the latter, which can produce false positives and false negatives due to the single stim regression) significantly reduces the accuracy even compared with the raw signal.

There is also the possibility that the increase in contrast achieved by Kalman regression will be more effective at increasing the classification accuracy for other types of stimuli or even for other classification features (for example, connectivity), in which systemic confounding signals are less likely to confound the classification accuracy.

### 4.4 Practical BCI Implementation

Even though we performed our demonstration as prerecorded data being streamed in real-time to the processing computer, the same pipeline could be used to perform online classification with a live user (as shown in Fig. 1). As regression techniques rely on knowing the timing of the stimulus onset, the task-presenting computer streams the stimulus (but not the stimulus class) to the processing computer. While a real BCI application might or might not have access to the explicit stimulus onset, other groups have proposed techniques for the automatic identification of the stimulus, either by additional measurements (such as eye tracking combined with machine object recognition^6^) or by statistical analysis of the data.^50^

As shown in Figs. 8 and 9, the classification accuracy of the fNIRS response only truly reaches above chance level around 7 s after the stimulus onset. This lag time is a known limitation for fNIRS BCI, and thus some alternatives have been proposed. For example, it has been proposed that hybrid EEG and fNIRS BCI could be implemented, which would combine the speed of the EEG response with the additional accuracy provided by fNIRS.^30^^,^^51^^,^^52^ Furthermore, not all real-time applications are time sensitive at the subsecond level: there have been some proposed applications such as passive BCIs^53^ or neuroergonomics that would accommodate a human user in the case of certain mental states (such as tiredness of extreme excitability), to prevent accidents, increase engagement, or provide feedback. Applications such as these can usually afford a delay of a couple of seconds before making a decision.

Our pipeline performed calculations for 24 simultaneous Kalman filters (one for each channel), each processing 400 samples per second. High-density fNIRS systems (with hundreds of channels) are becoming more common, and we expect the processing time to scale linearly with number of channels and sampling rate. This is offset by the fact that commercial and high density systems tend to have much lower sampling rate, closer to 1 Hz, requiring one to process less samples per channel. In addition, the multiple Kalman filters running in parallel for each channel are compatible with GPU/parallel computing (which we did not employ as it was not needed).

## 5 Conclusion

We demonstrated the real-time regression of fNIRS signals using a Kalman filter on a standard desktop PC. The signals were composed of 24 long separation and two short separation fNIRS channels and one accelerometer (with three axes). Temporally embedded short separation channels and accelerometer signals were used as input for the tCCA-based generation of nuisance regressors. The signals were processed at a rate of 400 samples per second by our real-time pre-recorded pipeline. Our online pipeline proves the feasibility of processing high data rate for a typical amount of fNIRS channels in real-time while at the same time calculating composite, temporally embedded regressors. The signals processed with the Kalman filter tuned with our resting data strategy showed a selective reduction in physiological noise as well as reduced high frequency noise, resulting in a higher CNR ratio for the HRF compared to nonregressed signals. CNR is generally lower for single wavelength regression; however, the CNR improvement produced by multimodal Kalman filter still increases the classification accuracy for the signals at 730 nm. These results could have implications in applications such as wearable BCI, where real-time processing is a requisite.

## 6 Appendix: Kalman Filter Algorithm and Tuning Strategies

### 6.1 Interpretation of the State Domain Model for HRF Regression

Equation (4) represents the state domain model used for Kalman HRF regression. x[n] is a vector containing the regression coefficients of the linear mixing model. In our implementation, x[n] contains the coefficients of the (40) Gaussians modeling the HRF, a drift term, and two regression coefficients for the tCCA regressors obtained from the accelerometer and short separation channels. In this model, the expected value of the state of the system will be equal to its initial state, but its variance will grow over time proportionally to the covariance matrix Q of the process noise w[n]. Thus, after n time steps, we expect the variance of the j’th regression coefficient to have increased by $nQ_{jj}$. The fNIRS measurement y[n] will be contaminated by the measurement noise v[n].

### 6.2 Kalman Filter Algorithm

The discrete-time Kalman filter is a recursive algorithm that finds an estimate of the state of a noisy system from its measurements based on a state-domain model. At every time step, the algorithm performs a prediction and an update step. The prediction step consists of the two equations:

x^[n|n−1]=x^[n−1|n−1]P^[n|n−1]=P^[n−1|n−1]+Q[n], (6)

where the hat on the variables represents an estimate of the variable as opposed to the true value of the variable. The notation x^[a|b] should be interpreted as “the estimation at time a with the information we had at up to time b.” The quantity P^ is the estimated error covariance matrix. The error covariance matrix is defined as P[n|n−1]=cov(x[n]−x^[n|n−1]). Thus, P^ is an estimate of how far the estimated state of the system (the regression coefficients) is from the real state of the system. The update step consists of the following equations:

K[n]=P^[n|n−1]C′[n](C[n]P^[n|n−1]C′[n]+R[n])−1P^[n|n]=P^[n|n−1]−K[n]C[n]P^[n|n−1]x^[n|n]=x^[n|n−1]+K[n](y[n]−C[n]x^[n|n−1]). (7)

-*The expression K[n] is the “optimal Kalman gain.” The term y[n]−C[n]x^[n|n−1] is known as the “innovation.” As the Kalman gain becomes smaller when the measurement noise R[n] is large, the algorithm “trusts” new measurements less when the measurement noise is large.

The variables x^[k|k] and P^[k|k] are the “state of the Kalman filter at time k.” To make the optimal prediction of x, good estimates of Q and R are required. Priors (P^[0|−1], x^[0|−1]) need to be provided to the algorithm to perform the initial update step. A good estimate of the priors will increase the convergence speed of the filter. A proper selection of the noises and the priors is called “tuning the Kalman filter.”

### 6.3 Kalman Filter Tuning

The correct initializations of the Kalman state broadly determines how fast the algorithm converges, and an appropriate estimate of the noise properties yields a better solution. In general, the parameters are channel specific. We propose that most of the tuning parameters can be estimated from a resting time series. The parameters estimated from the resting series can be used in the analysis of the experimental runs. The tuning parameters are specific to the subject, fNIRS hardware, optode placement, and operating conditions. The resting time series used in this work had a duration of 120 to 300 seconds per subject.

If we assume the HRF, the drift term and the tCCA regressors to be uncorrelated, then we can estimate the Kalman parameters for each:

P^[0|−1]=[PHRF000Pdrift000PtCCA]Q=[QHRF000Qdrift000QtCCA]. (8)

Similarly, x^[0|−1]=[xHRF,xdrift,xtCCA]T. We are not indicating the time dependency of $P_{j}$ and $x_{j}$ to simplify the notation and tuning only requires finding them for [0|−1]. The parameter R is independent of the three regression subcomponents (HRF, drift, and tCCA). The subparameters to find are summarized in Table 3.

**Table 3:** Kalman parameters to be tuned per channel.

Parameter	HRF subcomponent	Drift subcomponent	tCCA subcomponent
x^[0|−1]	$x_{\mathrm{HRF}}$. Determines the expected shape of the HRF for a given channel, subject, and stimulus	$x_{\text{drift}}$. Initial drift of the signal	$x_{\mathrm{tCCA}}$. Expected contribution of each tCCA regressor component to the signal (physiology, motion, etc.)
P^[0|−1]	$P_{\mathrm{HRF}}$. Expected initial covariance of the error between the expected HRF coefficients and the real HRF coefficients	$P_{\text{drift}}$. Expected initial variance of the error between the expected initial drift and the real initial drift	$P_{\mathrm{tCCA}}$. Expected initial covariance of the error between the estimated contribution of the tCCA components and their real contribution
Q	$Q_{\mathrm{HRF}}$ Covariance matrix of the random drift of the HRF coefficients	$Q_{\text{drift}}$. Variance of the drift/offset term	$Q_{\mathrm{tCCA}}$. Covariance matrix of the drift of the contribution of each tCCA component to the overall signal
R	Variance of the noise affecting the fNIRS measurements (instrument related). R is a scalar, and it is independent of the regression components.		

In the following sections, we will detail our tuning strategies for each of the subparameters.

#### 6.4 Initialization of the Estimated State Vector

The initialization that minimizes the initial error is x^[0|−1]=E[x[0]]. This requires having previous knowledge of the shape of the HRF and the contributions of the tCCA components. An alternative is to initialize it as zero, to not bias it to a particular solution. For this reason, we initialized the HRF components of the state vector $x_{\mathrm{HRF}}$ as zeros, as we do not know, *a priori*, how the HRF is going to look like. Similarly, the drift term, $x_{\text{drift}}$, was set to zero at t=0.

$x_{\mathrm{CA}}$ can be determined from the resting data. If the resting data were sampled for a long enough time, and if we can assume there is no strong neural component in the resting data, then the only components of the linear mixing model are the drift term and the tCCA components. We can then perform a least-squares estimation of the tCCA regression components by performing the following operation:

x^tCCA=UtCCA+yrest, (9)

where $U_{\mathrm{tCCA}}^{+}$ is the Moore–Penrose pseudoinverse of the matrix $U_{\mathrm{tCCA}}$. Each column of $U_{\mathrm{tCCA}}$ is a different tCCA regressor time series (calculated from the resting data) represented as a column vector. $y_{\text{rest}}$ is the resting time series. Then, x^tCCA will contain a least-squares estimate of the tCCA regression coefficients. This calculation needs to be done for each channel. If the acquisition for the resting data is long enough, we can expect x^tCCA=E[xtCCA].

In addition, for the implementation of the Kalman filter for classification purposes, it is necessary to reset the HRF components of the state vector at each stimulus onset. Not doing so would make the previous stronger responses to bias the estimation of the future responses. The drift and tCCA components are not reset as they are assumed to not change much between stimulus types, and they are expected to get better over time as more data are processed.

#### 6.5 Measurement Noise R

This could be calculated from the fundamental properties of the fNIRS hardware,^54^ but it can also be estimated from the power spectrum of the resting data. For this work, we calculated the PSD function of each resting data channel using Welch’s method (with default parameters). We assume that all the noninstrumental noise (motion, drift, physiology) is mostly confined to low frequencies. We set a threshold of 8 Hz for the limit of physiological noise and estimate the noise density in the band from 8 Hz to one half of the sampling frequency (100/2 Hz) by integrating the noise and normalizing by the bandwidth used to calculate it. We then multiply by the total bandwidth of the system to get an estimate of the physiological noise:

$$R\approx \frac{\frac{f_{s}}{2}}{\frac{f_{s}}{2}-8}\int_{8}^{\frac{f_{s}}{2}}\mathrm{PSD}(f)df.$$ (10)

This expression produces one scalar per channel. The PSD needs to be calculated for the data in the space that will be used for regression (for example, in the concentration space if the regression will be performed in the concentration space). In general, R was in an order of magnitude of 1e-11 $M^{2}$ (concentration space).

#### 6.6 Process Noise Covariance Matrix Q

In general, Q is not expected to be a diagonal matrix. The diagonal elements of Q represent the increase of variance of the regression coefficients at each time step. The off-diagonal elements represent the covariance between regression coefficient drifts. For example, $Q_{\mathrm{tCCA}}$ would be

$$Q_{\mathrm{tCCA}}=[\begin{array}{ccc} \mathrm{var}[\alpha_{1}] & \mathrm{cov}(\alpha_{2},\alpha_{1}) & \ldots \\ \mathrm{cov}(\alpha_{1},\alpha_{2}) & \mathrm{var}[\alpha_{2}] & \ldots \\ \ldots & \ldots & \ldots \end{array}],$$ (11)

where $\mathrm{var}[\alpha_{i}]$ represents the increase of variance at each time step for the i’th tCCA regression coefficient. The terms $\mathrm{cov}(\alpha_{i},\alpha_{j})$ represent the covariance in the drift of the i’th and j’th regression coefficients.

$Q_{\mathrm{HRF}}$ is the covariance matrix of the drift of the HRF shape. We can set $Q_{\mathrm{HRF}}$ as a zero matrix if we assume the shape of the HRF is fixed for a given stimulus.

We can estimate the variance of the drift $Q_{\text{drift}}$ from the resting data. We expect the drift to accumulate a variance of $kQ_{\text{drift}}$ after k samples. Thus, we estimated it by dividing the resting time series in J subintervals $y_{\text{rest}}^{j}[n]$ each with a length of k-samples. Then, we subtract the initial value from each respective partial time series to get ${\overset{˜}{y}}_{\text{rest}}^{j}[n]$:

$${\overset{˜}{y}}_{\text{rest}}^{j}[n]=y_{\text{rest}}^{j}[n]-y_{\text{rest}}^{j}[0].$$ (12)

We then calculate the variance of the J final values of each subtracted time series. That is $\mathrm{var}({\overset{˜}{y}}_{\text{rest}}^{j}[k])$. We expect this value to approximate $kQ_{\text{drift}}$. Thus, Q^drift=var(y˜restj[k])/k.

Similarly, for $Q_{\mathrm{tCCA}}$, we divide the resting data in J subintervals. After that, we calculated the tCCA filter and regressors for each subinterval. Then, for each subinterval j, we calculate the least-squares estimation of the tCCA regression coefficients for the subinterval:

x^tCCAj=UtCCAj+yrestj, (13)

where $y_{\text{rest}}^{j}$ is the j’th subinterval of the resting time series. $U_{\mathrm{tCCA}}^{j+}$ is the pseudoinverse of the tCCA regressors calculated for the corresponding subinterval $y_{\text{rest}}^{j}$. x^tCCAj are the estimated tCCA regression coefficients for the subinterval. We then calculate the variance of the J estimates of each coefficient and set:

QtCCA≈diag(var(x^tCCAj)), (14)

where the operator diag converts a vector into a diagonal matrix in which the main diagonal is the input vector.

There are several limitations for this calculation. First, it is desirable to get as many subintervals as possible to get a better approximation of the variance, but the tCCA calculation algorithm will fail if the subintervals are too short due to badly conditioned matrices, usually if they are shorter than 30 s. For this calculation, we divided the resting data in five subintervals of ∼30  s. This estimation ignores potential covariances between tCCA coefficients.

#### 6.7 Initialization of the Covariance Matrix of the Error P

P is defined as

P[a|b]=cov(x[a]−x^[a|b]), (15)

where x[a]−x^[a|b] is the error in the estimation of the state at t=a given the information known up to t=b (with a≥b). In general, we do not know the true state of the system, so the Kalman filter only calculates an estimate of P. If we initialize the state vector x^[0|−1] as zero, then Eq. (15) becomes:

P[0|−1]=cov([0]). (16)

Assuming again independence between HRF, drift, and tCCA, the approximation of P can be done by estimating the covariance matrix of the error of each of the three subcomponents. The initialization of $P_{\text{drift}}$ is straightforward, with $P_{\text{drift}}=Q_{\text{drift}}$. Similarly, we will initialize $P_{\mathrm{tCCA}}$ as $Q_{\mathrm{tCCA}}$.

The initialization of $P_{\mathrm{HRF}}$ requires knowing the HRF regression coefficients to calculate their covariance matrix. In general, we do not know this especially when dealing with a classification problem. For this reason, in the case of the real-time Kalman for the classifier, we initialized $P_{\mathrm{HRF}}$ as an identity matrix scaled by 5e-12 $M^{2}$ (equivalent to a standard deviation of 2.23  μM), or 5e-3 when processing the data in the optical density space. We determined this value as optimal with a synthetic data approach, by varying the scaling factor until the RMSE was minimized. However, a truly optimal initialization would initialize each component of the matrix independently and would include off-diagonal elements.

A different way to calculate P involves performing a second Kalman regression pass. In the first pass, we initialized P with the value mentioned in the previous paragraph. At the end of each run ($n=n_{f}$), we have estimated a final state vector x^s[nf] for each subject and channel, where the subscript s represents the subject number. With that, we can calculate an approximation of P given by:

P≈cov([x^1[tf],x^2[tf],x^3[tf],…,x^S[tf]]), (17)

where S is the total number of subjects and the term [x^1[tf],x^2[tf],x^3[tf],…,x^S[tf]] represents a matrix formed by arranging the (column) state vectors of each subject as a matrix. Then, this approximation of P is used as the initialization P[0|−1] for a second pass of regression. We observed great improvements in the overall performance of the regression in terms of RMSE for the augmented dataset when using this approach. The results in Fig. 5 used this approach. However, this approach cannot be used for the classification of the finger-tapping data as it cannot be determined in real time and only works if there is only one type of stimulus response.

For the classification regression, the HRF component $P_{\mathrm{HRF}}$ was reset at each stimulus onset to prevent previous stimuli from biasing future stimuli response estimations. The components of P for drift and tCCA were not reset.

#### 6.8 Summary of Tuning Process

Table 4 summarizes the proposed Kalman filter tuning from the resting dataset and the custom functions used to estimate each parameter. The functions can be accessed at a GitHub repository available at: https://github.com/BUNPC/KalmanTuning.

**Table 4:** Summary of Kalman tuning process.

Step	Output variable	GitHub function
Read raw resting data Y_raw and auxiliary data AUX	Yraw, AUX	—
Convert Y_raw to desired space (OD or Hb)	Y	—
Calculate measurement noise R from Y	R	estMeasNoise
Calculate tCCA regressors and filter from Y and AUX	REG	rtcca
Calculate tCCA coefficients with least squares pinv(REG)*Y	x_tCCA[0]	—
Set HRF and drift components of x [0] to zeros	—	—
Estimate drift component of Q	Q_drift	estQdrift
Estimate tCCA component of Q	Q_tCCA	est_tCCA_Q
Set HRF component of Q to zeros	—	—
Set P_drift = Q_drift and P_tCCA=Q_tCCA	—	—
Set HRF components of P to semiarbitrary value 5e-12*I [$M^{2}$] where I is the identity matrix (for Hb space) or 5e-3*I for OD space	P	—

Note: pinv represents the Moore–Penrose inverse
